# Multi-locus phylogenetic analysis reveals the pattern and tempo of bony fish evolution

**DOI:** 10.1371/currents.tol.2ca8041495ffafd0c92756e75247483e

**Published:** 2013-04-16

**Authors:** Richard E. Broughton, Ricardo Betancur-R., Chenhong Li, Gloria Arratia, Guillermo Ortí

**Affiliations:** University of Oklahoma; The George Washington University; Shanghai Ocean University; Courtesy Research Professor and Associated ResearcherUniversity of Kansas; The George Washington University

## Abstract

Over half of all vertebrates are “fishes”, which exhibit enormous diversity in morphology, physiology, behavior, reproductive biology, and ecology. Investigation of fundamental areas of vertebrate biology depend critically on a robust phylogeny of fishes, yet evolutionary relationships among the major actinopterygian and sarcopterygian lineages have not been conclusively resolved. Although a consensus phylogeny of teleosts has been emerging recently, it has been based on analyses of various subsets of actinopterygian taxa, but not on a full sample of all bony fishes. Here we conducted a comprehensive phylogenetic study on a broad taxonomic sample of 61 actinopterygian and sarcopterygian lineages (with a chondrichthyan outgroup) using a molecular data set of 21 independent loci. These data yielded a resolved phylogenetic hypothesis for extant Osteichthyes, including 1) reciprocally monophyletic Sarcopterygii and Actinopterygii, as currently understood, with polypteriforms as the first diverging lineage within Actinopterygii; 2) a monophyletic group containing gars and bowfin (= Holostei) as sister group to teleosts; and 3) the earliest diverging lineage among teleosts being Elopomorpha, rather than Osteoglossomorpha. Relaxed-clock dating analysis employing a set of 24 newly applied fossil calibrations reveals divergence times that are more consistent with paleontological estimates than previous studies. Establishing a new phylogenetic pattern with accurate divergence dates for bony fishes illustrates several areas where the fossil record is incomplete and provides critical new insights on diversification of this important vertebrate group.

## Introduction

The evolutionary history of bony vertebrates remains an outstanding problem in the Tree of Life. Osteichthyes, the group inclusive of ray-finned fishes (Actinopterygii) and lobe-finned fishes plus tetrapods (Sarcopterygii), contains all extant vertebrates except chondrichthyans and agnathans, yet our understanding of phylogenetic patterns and divergence times of many early-branching lineages remains incomplete. Phylogenetic resolution is vital for understanding vertebrate biology, including recent investigations of genome duplications 1 2, development and evolution of appendages 3, diversification rates associated with habitat preferences 4 5 6, reproductive systems 7, sensory and communication systems 8 9, hormone receptors 10 11, sodium channels 12, and developmental genes and regulatory elements^13^ ^14^ . However, sarcopterygian relationships are notoriously contentious 15 16 17 18 and much of the current view of ray-finned fish evolution remains based on morphological studies conducted nearly four decades ago 19 20 21. Resolving the phylogenetic branching order close to the base of the fish tree has been particularly challenging. Over the past 30 years numerous studies using morphological and molecular data have yielded conflicting results, contributing to persistent phylogenetic and taxonomic uncertainty (see 22 23 24 25 26 27 28 29 30). Although recent studies 31 32 have employed larger character sets to address some of these issues, their data sets are restricted to ray-finned fishes and do not include all lineages relevant to addressing relationships among bony fishes.

Difficulty in resolving phylogenetic relationships of bony fishes is due in large part to the ancient age but paucity of recent species in many lineages that diverged early from the main stem of the phylogeny. Among these, species richness is highly skewed with only 18 living species of Polypteriformes (reedfishes), 30 species of sturgeons and paddlefishes (Acipenseriformes), 7 species of gars (Lepisosteiformes ), and a single species of bowfin (Amiiformes), relative to at least 30,000 living species of teleosts 24. Similarly, among sarcopterygians there are 7 living species of lungfishes (dipnoans) and 2 living species of coelacanths (actinistians), compared to roughly 29,000 living tetrapods 33. The limited extant diversity in most of these groups is of relatively recent origin due to high rates of extinction among the early lineages. Consequently, with the exception of teleosts and tetrapods, the pattern of vertebrate phylogeny close to the base of the tree is one of very long phyletic branches that lack intervening divergences leading to extant descendants.

Although there is extensive representation of extinct lineages in the fossil record, including one of the largest and most diversified actinopterygian groups, the “palaeonisciforms”, these taxa remain poorly known 28. The fossil record of the 5 extant actinopterygian groups is comparatively much younger than that of extinct actinopterygians, with the oldest extinct forms known from the Late Silurian, whereas levels of molecular divergence suggest the extant groups arose in the Devonian 22 27 30. In Sarcopterygian lineages of Paleozoic age, a converse situation exists where fossil taxa (e.g., Porolepiformes, Osteolepiformes, Panderichthyida) are more closely related to tetrapods than are extant taxa 34. In both groups, fossil material required to confidently resolve deep divergences remains limited.

We are thus faced with a difficult phylogenetic problem, where brief periods of diversification caused short internodes deep in the tree followed by long periods of effective anagenesis (more accurately, cladogenesis with pervasive extinction) creating long terminal branches. Under this scenario, the majority of character variation arose after the major lineages were established, while few informative characters (synapomorphies) evolved within the temporal window between divergence events. Most morphological characters may thus have arisen outside the diversification window and be ancestral to many lineages (symplesiomorphies) or derived but unique to single descendant lineages (autapomorphies). Regarding molecular data, this scenario has been described as “the bane of the molecular systematist” 35, wherein the number of phylogenetically informative substitutions on stem branches is greatly outnumbered by homoplastic substitutions on the long terminal branches creating ambiguous or misleading phylogenetic signal 36. Because extant taxa of intermediate age that could bisect long branches do not exist in the present case, one of the few viable options to this long-branch problem is to assemble a large multilocus dataset to increase the potential of sampling of relatively rare synapomorphies on the short deep branches.

The sister group relationship between Sarcopterygii and Actinopterygii is well established. However, many alternative phylogenetic hypotheses have been proposed for the coelacanth-lungfish-tetrapod trichotomy as well as for actinopterygian and basal teleost divergences. Although it appears likely that polypterids are the sister group of all extant actinopterygian diversity 28 29 37 38 39 40 41 42, previous hypotheses linking Polypteriformes to Sarcopterygii have not been rigorously tested with a comprehensive data set (but see 39). There is little doubt that Acipenseriformes, Lepisosteiformes, Amiiformes and Teleostei are each monophyletic, yet their relationships remain unsettled. Acipenseriformes is conventionally recognized as the living sister group to the Neopterygii (Lepisosteiformes + Amiiformes + Teleostei) 23 24 27, although some molecular studies have suggested an “ancient fish” clade where Acipenseriformes, Lepisosteiformes and Amiiformes form a monophyletic group sister to Teleostei 29 39. Within the Neopterygii, there has been considerable disagreement on the identity of the sister group to teleosts, variously proposed as Amiiformes or Lepisosteiformes alone or as a monophyletic group containing both 19 43 44. Recent studies have suggested the validity of Holostei (Lepisosteiformes plus Amiiformes) based on molecular and morphological evidence 31 45 46.

The monophyly of the crown-group Teleostei is supported by many morphological characters 47 48 49 50 51 and is consistently recovered in molecular studies. Four major extant teleostean lineages include: Osteoglossomorpha (bony tongues, mooneyes and elephantfishes), Elopomorpha (tarpons, bonefishes, eels and relatives), Otomorpha (a large group containing Clupeiformes [shads and herrings] plus Ostariophysi including [minnows, catfishes, characins and relatives]), and Euteleosteomorpha (all remaining teleosts) 24. Otomorphs have been generally recognized as the sister group of euteleosts 48 52 53 54, whereas the conventional view is that osteoglossomorphs are the first diverging teleost lineage 20 55 56. However, accumulating morphological evidence suggests that elopomorphs may be the sister group to all other teleosts 54 57 58.

Molecular dating of particular divergence events is contingent upon accurate reconstruction of the nodes to be dated. While Bayesian relaxed clock methods (e.g., 59) accommodate topological uncertainty, dates are nonetheless associated with specific inferred divergences (nodes) on the tree. Previous molecular date estimates for divergence of fish lineages have varied widely, due in part to the variety of recovered topologies, but also due to a limited number of molecular loci, incomplete taxon sampling, and fossil calibrations that are limited in number, of uncertain age, or of uncertain taxonomic identity. In addition, different approaches to accommodate variation in evolutionary rates among lineages have been applied. For example, date estimates for the Actinopterygii-Sarcopterygii split have ranged from 476 to 423 million years ago (Ma) 20 30 60 61, while estimates of the age of the most recent common ancestor of teleosts span 376 to 185 Ma 22 27 30 31 61 62 63.

To provide for a more robust hypothesis of osteichthyan phylogeny and more accurate times of diversification, we conducted the first comprehensive analysis of bony fish evolution. We generated a data set of nearly 20,000 nucleotides from 21 loci from 61 species representing all major extant osteichthyan lineages plus chondrichthyan outgroups. Divergence dating employed an original set of 24 fossil calibrations that are chronologically proximal to focal divergence events. The resulting phylogenetic hypothesis and evolutionary time scale provide new insights on fish diversification and establish a framework for understanding their many evolutionary innovations.

## Materials and Methods

**Taxa** **** **and** **** **sequencing**

Sequences of 20 nuclear genes and 1 mitochondrial gene (Table 1) were obtained for 61 species that were selected to be broadly representative of the major actinopterygian and sarcopterygian lineages plus chondrichthyan outgroup taxa. We sequenced all loci from all taxa except where sequences were available in the NCBI or Ensembl databases. Single exons were sequenced from each nuclear gene (Table 1). Sequences of *rag1* and *rag2 *have been previously used in phylogenetic studies e.g., 64. The remaining 18 nuclear loci were developed as part of the Euteleost Tree of Life Project (www.fishtree.org) using a genomic screen method described by Li et al. 37. This compared the *Danio rerio* and *Takifugu rubripes* genomes to identify single-copy genes with long exons (>800 bp) and divergence levels suggesting they evolve at rates appropriate for phylogenetic resolution of the present taxa. The mitochondrial 16S ribosomal gene was sequenced from 11 species with the remainder acquired from GenBank.

**Table 1:** Gene loci used in this study.

Symbol	Name (NCBI gene ID *Danio rerio*)	Aligned Length
*apc2*	Adenomatosis polyposis coli 2 (565771)	798
*btbd7*	BTB domain containing 7 (568372)	852
*enc1*	Ectodermal-neural cortex (with BTB-like domain) (327531)	810
*ficd*	FIC domain containing (334648)	747
*gtdc2 * [glyt]	Glycosyltransferase-like domain containing 2 (497644)	888
*gpr85 * [sreb2]	G protein-coupled receptor 85 (793299 )	1071
*kbtbd4*	Kelch repeat and BTB (POZ) domain containing 4 (393178)	627
*kiaa-l*	Leucine-rich repeat and WD repeat-containing protein, KIAA1239-like (562320)	975
*myh6*	Myosin, heavy polypeptide 6 (386711)	1032
*panx2*	Pannexin 2 (557828)	783
*plagl2*	Pleiomorphic adenoma gene-like 2 (259255)	681
*ptchd4* [ptr]	Patched domain containing 4 (564097)	705
*rag1*	Recombination activating gene 1 (30663)	1632
*rag2*	Recombination activating gene 2 (30658)	1569
*ryr3*	Ryanodine receptor 3 (561350)	834
sacs-l	Sacsin-like (558150)	1137
snx33 [sh3px3]	Sorting nexin 3 (100001421)	705
tbcc-l [a3knt]	Tubulin-specific chaperone c-like (553378)	597
tbr1b [tbr1]	T-box, brain, 1b (58042)	687
zic1	Zic family member 1 (30096)	1050
mt-rnr2	16S RNA, mitochondrial (140506)	1817

Total DNA was extracted from ethanol preserved muscle samples with the DNeasy Tissue Kit (Qiagen), following manufacturer's instructions. Nested PCR was performed in successive reactions where a 100-fold dilution of the first reaction was used as template for the second reaction. Amplifications were performed in 25 μl volumes with 2.5 μl of 10X buffer , 2 μl dNTPs (200 nM of each), 1.25 μl each 10 mM primer, 0.25 μl Taq Gold polymerase (Invitrogen) or TopTaq (Qiagen), 2 μl template DNA (~100 ng), and 15.9 μl ddH2O. We employed a touchdown cycling program that included 5 cycles of primer annealing at 56°C, 5 cycles at 54°C, 5 cycles at 52°C, and 25 cycles at 50°C. After checking reaction success on 1% agarose gels, products were prepared for sequencing reactions enzymatically with ExoSap-It (USB Corp.) or via filtration with Multiscreen PCR 96 filter plates (Millipore Inc). Purified products were sequenced with Big Dye Terminator v 3.1 chemistry on a 3130xl Genetic Analyzer (Applied Biosystems). Contigs of individual loci were assembled and aligned with Geneious Pro 4.5 (Biomatters Ltd.). Few cases of apparent heterozygosity were observed but potential heterozygosity or ambiguous base calls were designated with standard IUB ambiguity codes. Alignment of protein gene segments was refined by codons with Mesquite v. 2.72 65. New sequences have been submitted to GenBank and the alignment file has been deposited in Dryad (http://dx.doi.org/10.5061/dryad.f1t15).

**Phylogenetic** **** **analyses**

Evolutionary model selection was performed for the concatenated alignment using MrAIC 66, where the GTR+G+I was identified as the best fit to the data. To assess heterogeneity in phylogenetic signal among loci, each gene segment was analyzed individually by maximum likelihood (ML) with RAxML v. 7.4.2 67. Analyses employed rapid non-parametric bootstrapping (100 pseudoreplicates) using GTRCAT (GTR substitution model with the CAT approximation for among site rate variation), followed by a search for the tree that yielded the ML under GTRGAMMA (GTR with a 4-class gamma model of among site rate variation). Resulting topologies were compared to each other and to that obtained from a concatenated unpartitioned data set. Individual gene trees were often poorly resolved but they were typically consistent with the tree from the concatenated analysis. Incongruent nodes were rarely obtained from more than one gene and typically had bootstrap proportions <60%. We therefore focused analyses on the concatenated dataset of all 21 genes.

Analyses of the combined data employed ML with RAxML and Bayesian analysis with MrBayes v. 3.2.1 68. All analyses assumed unrooted trees where the outgroup (chondrichthyans) was not assumed to be monophyletic. Analyses with RAxML employed search parameters described above for single genes but with 1000 bootstrap pseudoreplicates. Analyses were performed using two partition schemes: one with 4 data partitions (3 codon positions plus 16S), and one with 14 partitions (a combination of codon positions and individual genes plus 16S) as recovered by PartitionFinder under the BIC criterion 69. To explore potential effects of saturation at 3rd codon positions, we analyzed a dataset with all 3rd positions excluded (unpartitioned). These data schemes were also used in MrBayes with the GTR+G+I model and default settings for 10 million generations with 25% burn-in. Alternative approaches to analyze combined data based on “species trees” methods that account for gene-tree heterogeneity due to lineage sorting, e.g. 70 71 72 73, could not be applied to this data set due to missing data for some gene/taxon combinations (see Results).

**Dating divergence events**

A Bayesian relaxed clock method with an uncorrelated lineage-specific model of rate variation 59 implemented in BEAST v. 1.6.2 74 was used to estimate divergence times. Fossil calibrations (Appendix 1) were assigned to appropriate nodes using prior distributions according to degree of completeness or confidence of the fossil record for each case 75. Where minimum ages for specific nodes could be unambiguously determined based on the fossil record, these ages were used as hard minima in log-normal distributions with fossil-based soft maxima used to estimate the standard deviation of the distribution. In other cases (i.e., where there were apparent stratigraphic gaps in the fossil record), uncertainty was accommodated using uniform distributions with minimum-age offset values (see discussion in Appendix 1). We note that with multiple calibrations, calibration densities employed by BEAST do not reflect the actual marginal prior distributions of particular nodes. Although one or more "rogue" calibrations could have misleading effects on estimated dates, a practical solution to identifying these from among a large set of calibrations remains elusive. To model branching rates on the tree, a birth-death process was used for the tree prior with initial birth rate = 1.0 and death rate = 0.5. The substitution model was GTR+G with 4 rate classes and the data were partitioned into 4 categories with independent parameter estimation: the 3 codon positions for exons plus the ribosomal gene. Two replicate analyses were performed with BEAST, each run for 200 million generations, with the topology constrained to that recovered in phylogenetic analyses. Post-run analysis of MCMC log files was assessed using Tracer v. 1.5 and mixing was considered complete if the effective sample size of each parameter was >200. Tree files from the two runs were combined with the first 10% of trees from each run discarded as burn-in. The combined sample of trees was used to assess posterior probabilities, HPD node heights (dates), and credibility intervals. The results were visualized with FigTree 74.

## Results

**Phylogenetic analysis**

Sequences were generated from 20 nuclear exons and 1 mitochondrial rRNA gene yielding 19,997 aligned nucleotide positions. Data were obtained from 61 taxa, including 2 chondrithyans, 7 sarcopterygians and 52 actinopterygians representing all extant basal lineages. There were 12,161 variable positions of which 10,493 were parsimony informative. The resulting data set is 62% complete as not all genes could be obtained from all species (Appendix 2). All phylogenetic analyses obtained the same topology, illustrated in Figure 1, with corresponding support values in Table 2. This tree has broad support and all recognized taxonomic orders and other nominal groups were recovered as monophyletic. Although bootstrap support was somewhat lower for the lungfish + tetrapod clade and the Osteoglossocephalai clade in partitioned analyses, support for these nodes was strong where 3rd codon positions were excluded. This suggests that 3rd codon positions harbor substantial homoplasy in deep parts of the tree, but the effects of saturation are only evident where internal branches are very short and there are proportionately fewer historically consistent characters. For all other nodes, the use of different data partition schemes or exclusion of 3rd codon positions had negligible effects.

**Phylogenetic tree obtained from Bayesian analysis of concatenated data with 14 partitions.**
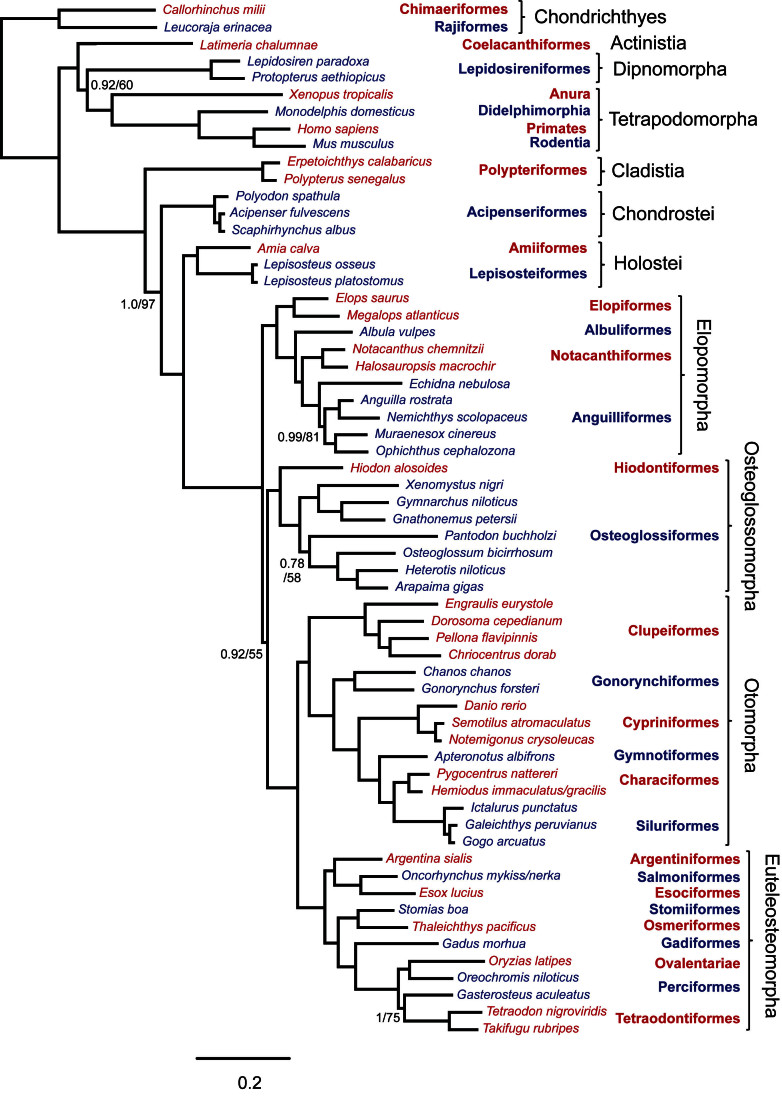
Topology of ML analysis was identical and branch lengths were similar. Support values are shown for Bayesian posterior probabilities (left of /) and ML bootstrap percentages (right of /) where at least one value was less than 1.0 or 100%. All other nodes had support values of 1.0/100. Red and blue color of taxon labels indicates inclusion in corresponding taxonomic order listed to the right. Scale bar is probability of nucleotide change.

**Table 2:** Support values from phylogenetic analyses and age data for selected branches.

Crown Clade	Bayesian PP*	ML Bootstrap*	Mean Age	95% HPD
Gnathostomata (= Root)			465.4	427.4 - 504.7
Osteichthyes	1.0, 1.0, 1.0	100, 100, 100	426.6	418.0 - 437.9
Sarcopterygii	1.0, 1.0, 1.0	99, 100, 100	409.3	407.0 - 413.8
Dipnotetrapodomorpha	0.84, 92, 0.99	58, 60, 96	375.0	350.3 - 398.3
Tetrapoda	1.0, 1.0, 1.0	100, 100, 100	335.1	330.0-345.1
Actinopterygii	1.0, 1.0, 1.0	100, 100, 100	384.3	375.0 - 398.4
Polypteriformes	1.0, 1.0, 1.0	100, 100, 100	38.7	10.8 - 75.1
Actinopteri	1.0, 1.0, 1.0	100, 97, 70	354.6	325.6 - 381.0
Chondrostei	1.0, 1.0, 1.0	100, 100, 100	132.5	125.0 - 147.3
Neopterygii	1.0, 1.0, 1.0	100, 100, 100	327.8	296.2 - 358.1
Holostei	1.0, 1.0, 1.0	100, 100, 89	268.6	246.0 - 303.2
Teleostei	1.0, 1.0, 1.0	100, 100, 100	283.5	258.9 - 313.0
Elopomorpha	1.0, 1.0, 1.0	100, 100, 100	215.2	172.1 - 259.6
Osteoglossocephalai	0.61, 0.92, 0.96	43, 55, 92	271.2	242.7 - 298.4
Osteoglossomorpha	1.0, 1.0, 1.0	100, 100, 100	230.9	197.5 - 260
Osteoglossiformes	1.0, 1.0, 1.0	100, 100, 100	189.2	148.5 - 230.2
Clupeocephala	1.0, 1.0, 1.0	100, 100, 100	245.4	217.3 - 273.7
Otomorpha (= Ostarioclupeomorpha)	1.0, 1.0, 1.0	100, 100, 100	225.9	197.6 - 256.0
Clupeiformes	1.0, 1.0, 1.0	100, 100, 100	140.4	90.5 - 192.1
Ostariophysi	1.0, 1.0, 1.0	100, 100, 99	195.5	166.1 - 226.3
Otophysi	1.0, 1.0, 1.0	100, 100, 96	164.0	131.8 - 197.1
Euteleostei	1.0, 1.0, 1.0	100, 100, 100	206.8	173.3 - 238.6
Acanthomorpha (in part)	1.0, 1.0, 1.0	100, 100, 100	158.1	128.2 - 188.4
				
* Support values for nodes in Fig. 1; listed from left to right for analyses with 4 data partitions, 14 data partitions, 3rd codon positions excluded (see text for descriptions).				

The phylogenetic hypothesis includes strong support for reciprocal monophyly of Actinopterygii and Sarcopterygii. Among sarcopterygians, coelacanths were the sister group to dipnoans plus tetrapods (i.e., dipnotetrapodomorphs). The position of the chondrichthyan outgroup node placed Polypteriformes as the earliest diverging group within Actinopterygii. The next actinopterygian group to diverge was the chondrosteans (Acipenseriformes). We obtained strong support for a monophyletic Holostei, rather than either Lepisosteiformes or Amiiformes alone, as the sister group to teleosts. Elopomorpha was consistently obtained as the earliest diverging teleost lineage. This is counter to the previously conventional view of Osteoglossomorpha as the earliest diverging teleost lineage. In our analyses, the Osteoglossocephalai clade was particularly well supported in analysis with 3rd codon positions excluded with a bootstrap percentage of 92. Osteoglossomorpha was sister to Clupeocephala (= Otomorpha plus Euteleosteomorpha). Elopomorpha was recovered as monophyletic, with successive branches leading to monophyletic Elopiformes, Albuliformes, Notacanthiformes and Anguilliformes, while within Osteoglossomorpha, Hiodontiformes was sister to monophyletic Osteoglossiformes. The recently hypothesized Otomorpha (=Ostarioclupeomorpha) was monophyletic and formed the sister group to the euteleosts.

**Dating lineage divergences**

The time tree for bony fishes is shown in Figure 2. Mean divergence times with 95% credibility intervals for major nodes are listed in Table 2. These are also illustrated in Figure 3 with comparison to two recent studies of ray-finned fishes that were based on multiple nuclear loci (estimates based on mtDNA were not included due to potential confounding effects of nucleotide saturation 76). Date estimates presented here suggest that divergences of major ray-finned fish lineages were considerably older than the oldest known fossils for their respective groups. Our estimate of 427 Ma for divergence of crown Osteichthyes places the origin of Sarcopterygii and Actinopterygii in the Middle Silurian, with the sarcopterygian crown group evolving in the Early Devonian (409 Ma) and the actinopterygian crown group evolving at the Middle-Late boundary of the Devonian (384 Ma). Actinopteri and Neopterygii then diverge successively at roughly 30-million year intervals (355 Ma and 328 Ma, respectively) in the Carboniferous (Mississippian epoch). Mean date estimates for basal actinopterygian divergences tend to be younger than those of other recent studies, although 95% probability distributions overlap in many cases (Figure 3). Initial divergence of crown group Teleostei appears to have occurred rapidly in the Early Permian (284 Ma), generating three major lineages, Elopomorpha, Osteoglossomorpha and Clupeocephala, in a span of 13 million years. However, no extant descendants emerge from these lineages until the Early Triassic (e.g., the earliest is crown Clupeocephala, 245 Ma). The most diverse teleost groups, Anguilliformes, Otophysi, and Acanthomorpha, did not begin to diversify until the Middle Jurassic.

**Timetree showing divergence times for fish lineages based on Bayesian UCLN analysis with 24 age constraints.**
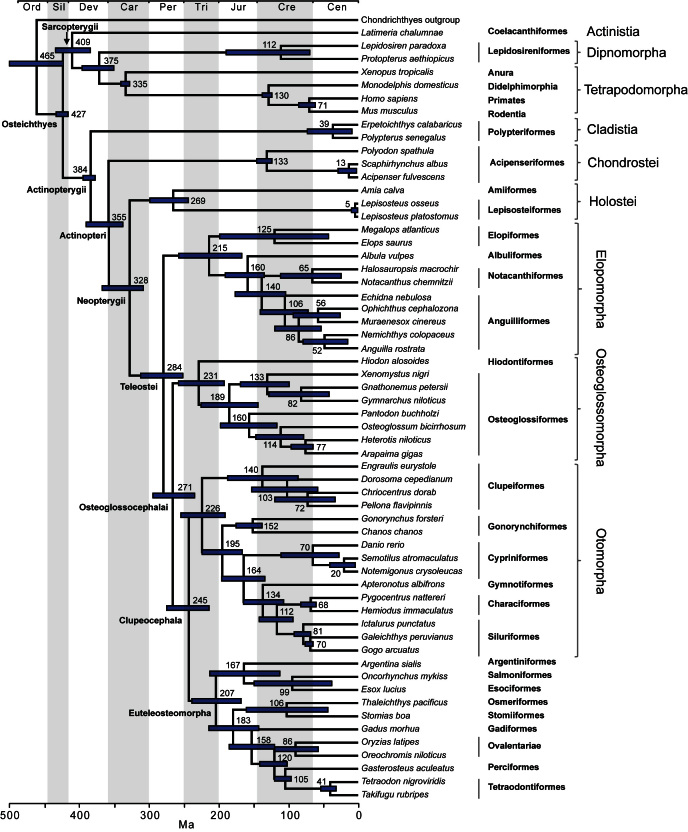
Mean estimates of divergence times are listed near nodes and bars indicate 95% credibility intervals.

**Comparison of mean (triangle) and 95% credibility intervals (horizontal bars) of divergence dates for fish taxa.**
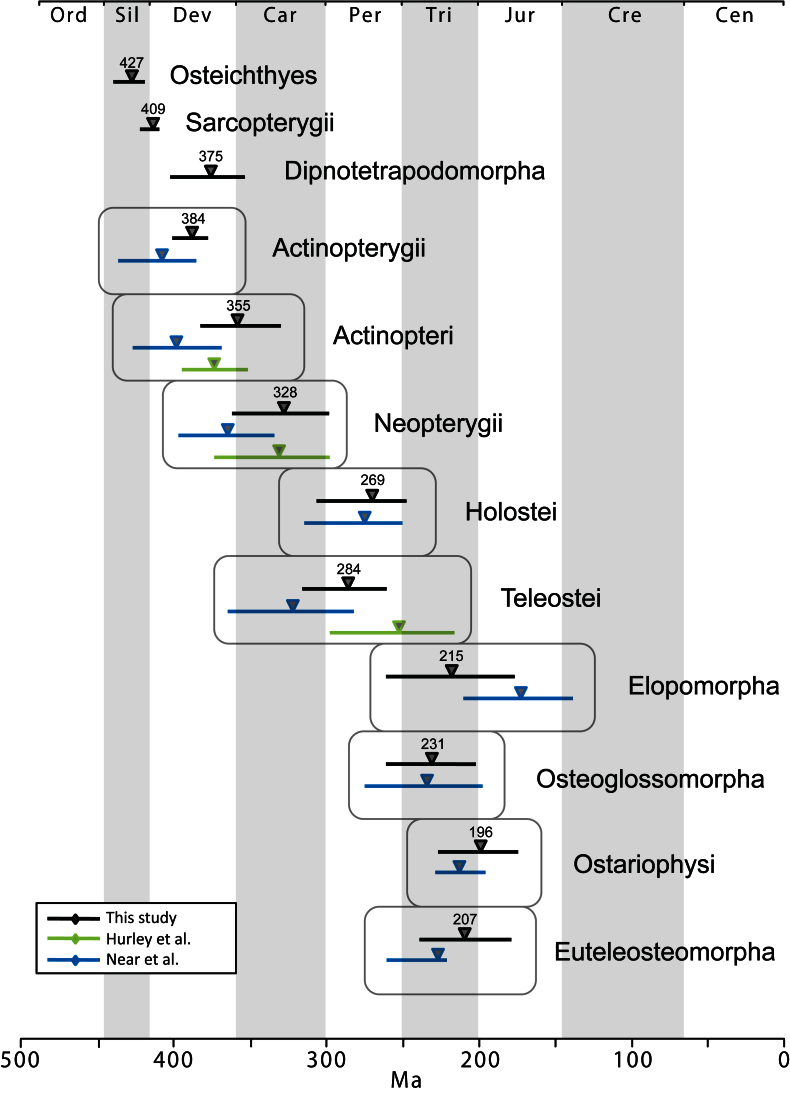
Black lines and mean dates are from this study, green are from Hurley et al.^27^ and blue are from Near et al.^31^ Where lines are absent, the particular date estimation was not performed in the corresponding study

## Discussion

Our analyses of ray-finned fish phylogeny employed 21 independent loci and broad taxon sampling within all lineages where multiple extant taxa exist. The remarkably strong node support suggests that the number and combination of loci used accurately capture phylogenetic signal for long phyletic branches emerging near the base of Actinopterygii. We note that in ML analyses using individual gene segments, few searches resulted in this exact topology and bootstrap support for nodes was often weak (<60%), yet collectively the data contribute to a well-resolved tree. Thus, combining many independent loci helps resolve difficult phylogenetic problems, where the additive phylogenetic signal of combined loci provides support for individual nodes and can overcome extensive but randomly distributed homoplasy. Although divergences near the base of Sarcopterygii and Teleostei were separated by exceptionally short time spans, it appears that the specific combination of genes sampled retain a sufficient number of historically consistent characters to resolve these two nodes. Whereas a recent study recovered a similar phylogenetic hypothesis for actinopterygian taxa, albeit using an assumed root and fewer loci (a subset of the loci included here) 31, our inclusion of sarcopterygian and chondrichthyan taxa and more than double the number of loci enable greater confidence in phylogenetic inferences, particularly with respect to basal osteichthyan and actinopterygian divergences. In addition, our novel fossil calibrations allow a new level of accuracy in dating divergence events in the bony fish tree of life.

**Divergence of Actinopterygii and Sarcopterygii**

Although the sister group relationship of Sarcopterygii and Actinopterygii is not controversial, the hypothesis that Cladistia (including Polypteriformes) is included in Actinopterygii has not been rigorously tested. Various characters including jaw, branchial arch, scale and limb morphology have suggested affinity of Cladistia with sarcopterygians 15, while other morphological 77 and molecular 38 39 characters are consistent with Cladistia forming the sister group to all other actinopterygians. Previously, few molecular studies (but see 38 39) have included the taxa necessary (including an outgroup) to formally test the phylogenetic position of Cladistia within Actinopterygii. Our results strongly confirm this relationship, supporting the division of actinopterygians into two clades of equal rank, Cladistia and Actinopteri. Our date estimate of 427 Ma for the sarcopterygian-actinopterygian split is slightly older than the fossil evidence would suggest. However, the fossil record of polypteriforms is very incomplete in comparison to other actinopterygians. This is an example of the fossil record being much younger than the phylogenetic position implied by both morphological and molecular data would indicate. The oldest polypteriform fossil records are from the Albian of Brazil 78 and the Cenomanian of Morocco (Kem Kem beds, Cenomanian) 79 80 (93.5-112 Ma). Thus, several hundred million years of polypteriform fossils are completely absent and the oldest occurrence grossly underestimates the necessary age of the lineage. Reviewing available fossil evidence, Benton et al. 81 estimated a hard minimum date for the split of Actinopterygii and Sarcopterygii as 416 Ma with a soft maximum of 422 Ma, although the latter is based on taxonomic assignments for which there is some uncertainty with respect to interpretation of *Andreolepis* and *Lophosteus* as early actinopterygians. Here, we consider the Late Silurian *Andreolepis* and as a stem osteichthyan 82 (despite some remaining doubt about its phylogenetic position), with the Late Silurian *Ligulalepis yunnanensis* as the oldest actinopterygian.

**Interrelationships of coelacanths, lungfishes and tetrapods**

The identity of the living sister group to Tetrapodomorpha has been controversial since the discovery of a living coelacanth in 1938. These relationships have received considerable attention, with many studies hypothesizing each possible resolution of this trifurcation. The pattern of relationships obtained in our analyses, with lungfishes and tetrapods forming a monophyletic group exclusive of coelacanths is consistent with several previous studies 16 17 83. However, an hypothesis of lungfishes plus coelacanths forming the sister group to tetrapods has been supported by morphological 34 84 85 and molecular data 60 86 87 , while a coelacanth plus tetrapod clade has also been hypothesized 18 88 89. Indeed, these taxa were recently described as an example of an extraordinarily difficult phylogenetic problem, where branches separating divergences are very short relative to terminal branches that are very long 35. This is perhaps further illustrated by the difference of our results with a recent phylogenetic study which obtained the coelacanth plus lungfish clade using 43 separate loci 87. However, the discrepancy may also be related to a smaller sample of taxa (7 in total) and/or the use of a less realistic evolutionary model (unpartitioned data) in that study 87.

The closer fossil relationship to tetrapods appears in the clade comprising (Osteolepiformes + (Elpistostegalia + Tetrapoda) (e.g. 89 90 91). The oldest known fossil coelacanth, *Euporosteus yunnanensis*, was recently described from the Early Devonian (Pragian) of China as morphologically similar to extant forms 92, but unfortunately stem-group coelacanths remain unknown. The oldest known stem dipnoan, *Diabolepis*, was also recovered in the Early Devonian (Lochkovian) of China 93. Our divergence date estimates of 409 Ma for the crown of extant Sarcopterygii is in agreement with these fossil ages. Moreover, the divergence of South American and African lungfishes estimated at 112 Ma is centered on the time interval for opening of the South Atlantic Ocean at 125-100 Ma 94, consistent with a vicariant explanation for their divergence.

**The sister group to teleosts**

Identity of the sister group to teleosts has long been problematic but is of key interest because teleosts exhibit many evolutionary novelties and understanding the ancestral condition of such traits requires a robust resolution of the sister group relationship. Our results support Holostei (Lepisosteiformes + Amiiformes) as the extant sister group to Teleostei. Historically, the hypothesis that the holosteans (Lepisosteiformes and Amiiformes) were closely related to teleosts was generally accepted 95 96 97. However, subsequent analyses suggested *Amia* 19 40 98 or *Lepisosteus* 43 alone shared a more recent common ancestor with Teleostei. The halecomorph hypothesis of *Amia* and teleosts forming a monophyletic group (e.g., 20 98) has been generally accepted for over 30 years. However, Arratia 44 reviewed several alternative hypotheses, finding that no particular pattern could be supported by a preponderance of the available morphological characters exhibited by both fossil and living taxa. More recently, Hurley et al. 27 recovered the Holostei when fossil data were included, but not when 4 nuclear genes were analyzed alone. Then, in a thorough reassessment of the lepisosteiform morphology, Grande 46 provided evidence for resurrection of Holostei. Consistent with molecular studies that provided additional evidence for this arrangement 31 38 45 61 63, our results provide compelling support for recognition of Holostei.

The present molecular date for crown neopterygians of 328 Ma is surprisingly similar to the 330 Ma estimate of Hurley et al. 27 based on their re-diagnosis of *Brachydegma* as a stem amiiform. *Brachydegma* was previously considered to be a paleoniscid 99 and Hurley et al.’s interpretation has recently been questioned 31. After review of the specimens by one of us (G.A.), we find that there is no support for *Brachydegma* as a stem halecomorph, in addition to the oldest crown group holostean and neopterygian, because *Brachydegma* lacks the synapomorphies of neopterygians, but it has paleonisciform characters. Given the extensive differences in calibrations between this study and Hurley et al. 27, the similar dates appear to be serendipitous convergence from different taxa and models of rate estimation. Our date of 284 Ma for crown teleosts is, however, significantly older than Hurley et al.’s 27 estimate of 250 Ma and much older than the minimum fossil age of about 216–204 Ma for *Pholidophorus latiusculus*, the oldest known teleost 49.

Many duplicated genes appear to have arisen in the ancestor of teleosts, suggesting a whole-genome duplication in that lineage 1 2. We specifically excluded duplicated genes to avoid potentially paralogous genes and so cannot comment directly on phylogenetic placement of the actual duplication event(s). However, based on the present dates, duplications in the teleost stem lineage should have occurred within the window of approximately 284–328 Ma. This range partially overlaps the older end of fossil-calibrated estimate using duplicated genes of 226–316 Ma 27, and partially overlaps the younger end of estimates based on assumed evolutionary rates of duplicated genes which place it older than 320 Ma 100 101 102.

**Relationships of early teleost lineages**

Within teleosts, our results place Elopomorpha as the earliest branching lineage among the teleosts, Osteoglossomorpha as the sister group to Clupeocephala. Otomorpha (=Ostarioclupeomorpha) was obtained as the sister group to the derived teleosts (Euteleosteomorpha). Recognition of the monophyly of Clupeiformes plus Ostariophysi has received broad acceptance, with the description of many morphological synapomorphies 51. However, divergence of Elopomorpha at the base of the teleosts is counter to the long-prevailing view that the Osteoglossomorpha represents the earliest branching teleost lineage 55 56, following Patterson and Rosen 20. Greenwood et al. 21 proposed three major divisions of teleosts: Division I Elopomorpha, Division II Osteoglossomorpha, Div III Ostariophysi + current Euteleosteomorpha. The divisions were presented as a basal trifurcation, leaving the branching order unspecified. Recent mtDNA analyses have supported the basal divergence of the lineage leading to extant osteoglossomorphs 52 61 62. The hypothesis of elopomorphs as the first diverging teleosts was proposed by Arratia based on the most comprehensive morphological studies including fossil and Recent teleosts to date 48 49 50 54 57 58. While other morphological studies have been consistent with this hypothesis 40 103 104 105, it was not broadly accepted (e.g., 106). A recent investigation of ray-finned fish diversification based on the single rag1 gene presented elopomorphs as the earliest branching teleosts without comment 22 , and this relationship was recovered in an analysis that focused largely on euteleosts 31. This appears to be another case where short times separating divergence events result in reduced phylogenetic signal. Indeed, this appears to be reflected in somewhat lower bootstrap support for the Osteoglossocephalai clade in our analyses, particularly where 3rd codon positions are included. Yet our consistent recovery of this relationship in all analyses, regardless of character inclusion status or partition scheme, suggest considerable confidence may be placed in this result.

**Classification of Actinopterygii**

The phylogenetic relationships presented here point to a revision of actinopterygian taxonomy. According to Nelson 24 the class Actinopterygii included the subclasses Cladistia, Chondrostei, and Neopterygii. The subclass Neopterygii *sensu* Nelson 24 following Patterson 19 included two main groups, the Halecostomi and Halecomorphi. The Halecostomi comprised taxa such as the fossil Semionotiformes and the extant Ginglymodi (e.g., Lepisosteiformes), whereas the Halecomorphi included among others, the fossil Parasemionotiformes, the extant Amiiformes and the division Teleostei. Our results demonstrate that actinopterygians should be divided into two clades, Cladistia and Actinopteri, with the latter including Chondrostei and Neopterygii. Holostei and Teleostei are thus two clades of equal rank within the subclass Neopterygii. This implies a new usage for the name Neopterygii. A similar classification was proposed by Wiley and Johnson 25 based on a revision of morphological synapormorphies from the literature. The Holostei that until 1973 was considered a subclass (based on Müller 107), has been absent from fish classifications for the last 40 years. Our data indicate that it is a valid clade that has been recovered in other recent molecular (e.g., 31 37 38 45 61, and morphological 46 108 studies. On the other hand, the Teleostei proposed by Müller 107 as a subclass, was more recently interpreted as a division of the Halecomorphi 19. Morphological characters supporting the monophyly of the Halecomorphi were provided by Grande 46, and characters supporting the monophyly of Teleostei were provided by Arratia 44 48 49 50. The Teleostei includes at its base a series of important and well documented fossil groups 44 48; the extant Elopomorpha stands as the earliest branching lineage of the large crown-group Teleostei, confirming previous phylogenetic hypotheses based on morphological evidence by Arratia 48 54 and others 104 105. The Elopomorpha should thus be included in the Elopocephalai that is the sister group of Osteoglossocephalai (Osteoglossomorpha plus Clupeocephala), where Clupeocephala includes Otomorpha (=Ostarioclupeomorpha) and the more advanced teleosts (the euteleosts) or Euteleosteomorpha 48. The classification scheme is fully developed in a companion paper where it is extended to all of bony fish diversity.

**Timescale of bony fish evolution**

Our dating analysis incorporated 24 fossil calibrations, including many novel calibrations from the recent paleontological literature, with 22 on basal osteichthyan branches. Our taxon sample is unique among recent studies, allowing direct estimation of divergence dates for crown Osteichthyes, Actinopterygii and Sarcopterygii, in addition to basal branches within those groups. This dense sample of directly relevant calibration points in combination with numerous independent loci provides a new level of accuracy in dating of osteichthyan divergence dates.

Our estimate of 427 Ma places the origin of sarcopterygians and actinopterygians in the Early Silurian. While this date is somewhat older than the oldest fossils that can be unambiguously assigned to each group, it is substantially younger than most previous molecular studies where it was directly estimated (e.g. 476 Ma 60). Crown Sarcopterygii diverged into coelacanth and dipnoans plus tetrapod lineages fairly rapidly at 409 Ma, whereas the most recent common ancestor of extant Actinopterygii did not appear until 384 Ma. Mean divergence dates suggest that actinopterygian lineages arose gradually over the next 100 million years or so with crown Teleostei appearing at approximately 284 Ma. Three major teleost stem lineages, Elopomorpha, Osteoglossomorpha and Clupeocephala, then arose rapidly in the Middle Permian. Through the Late Permian and Early Triassic, there are few cladogenic events but in the Late Triassic, crown groups Elopomorpha, Osteoglossomorpha, Otomorpha and Euteleosteomorpha all begin to diversify.

The timing of teleost diversification suggests conditions surrounding the end-Permian mass extinction significantly shaped the history of ray-finned fishes. Events of the Late Permian resulted in extinction of up to 96% of all marine species, including much of the fish diversity of that time 109. Complete lineages such as armored agnathans, placoderms, and acanthodians did not survive the Permian, while many sarcopterygian and some paleonisciform groups also went extinct. Prior to their demise, the diversity of such groups likely presented emerging teleosts with largely occupied niche space, thus limiting opportunities for diversification. In contrast, post-Permian environmental conditions appear to have been severely limiting for many forms of life and the prior extinction of much of the flora and invertebrate fauna contributed to reduced primary productivity and resource availability. The fossil record suggests these conditions persisted for up to 10 million years and that full biotic recovery did not occur until the Late Triassic 110 111. Only at this time did Elopomorpha, Osteoglossomorpha, Otomorpha and Euteleosteomorpha begin to diversify in a temporally close series of events (231-207Ma). Therefore, it appears that diversification of the major teleost lineages is closely associated with amelioration of the restrictive environmental conditions resulting from Late Permian events.

## Figures and Tables

**Tree showing branch locations of fossils used in dating analysis (Fig. S1).**
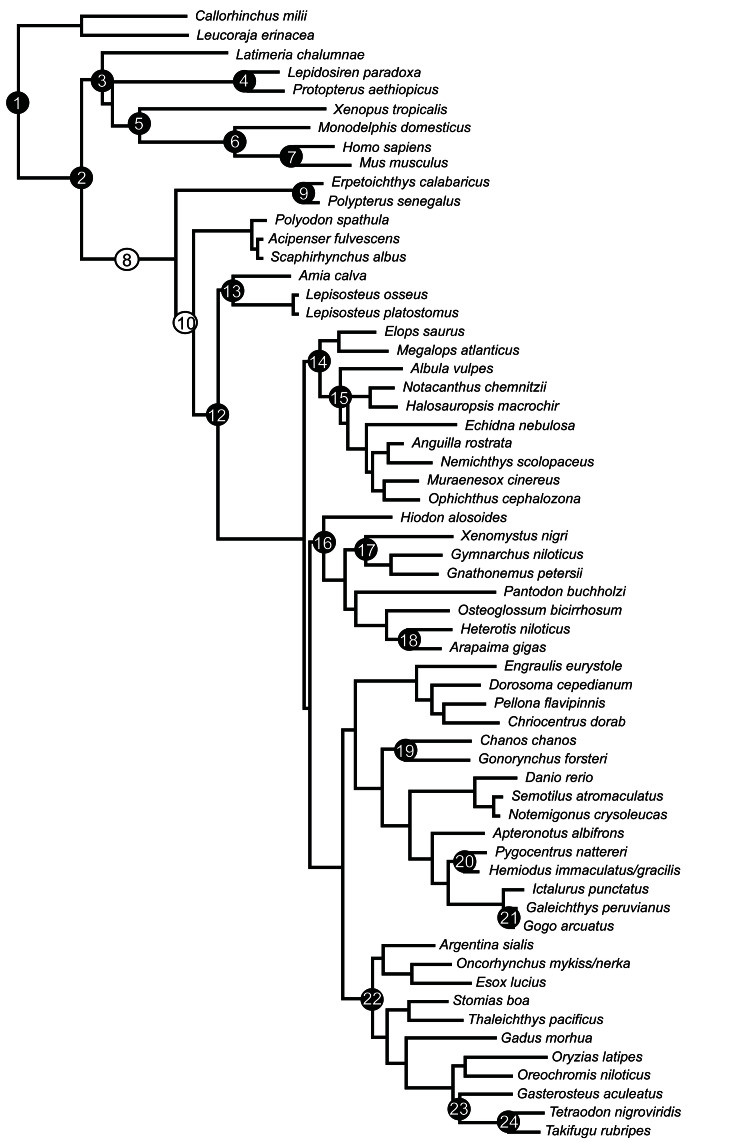
Numbers in circles correspond to numbered descriptions in text below. Black circles indicate crown calibrations; white circles indicate stem calibrations.

## Appendix 1

### Calibration points and age prior settings

This section provides details on fossil calibrations used for divergence time estimates. Hard lower bounds or minimum age reflect the youngest possible age interpretation of the fossils, rather than mid-point of age range (see [1]); soft upper bounds or maximum age reflect the oldest possible fossil age. Fossil calibrations were assigned prior distributions according to degree of completeness or confidence of the fossil record for each [2].  Where minimum ages appeared to be fairly secure these were used as hard minima in exponential distributions with fossil-based 95% soft maxima used to estimate the mean and standard deviation of the distribution.  In other cases there was evidence for predicting the existence of ghost fossil lineages, for which uniform distributions were used.  For example, although the gonorynchiform family Chanidae is deeply nested within ostariophysans, the oldest crown member of this family, † *Rubiesichthys gregalis * (Early Cretaceous (145.5-139 my; [3, 4]), is also among the oldest records for the Clupeocephala clade (i.e., Ostarioclupeomorpha + Euteleostei; sensu [5]). While † *Rubiesichthys * is slightly younger than other clupeocephalans, such as the euteleosts † *Orthogonikleithrus* *and* †*Leptolepides * (ca. 153–149 Ma [6]) and the stem ostariophysan † *Tischlingerichthys * (146.5-145.5 Ma [6]), its old age and nested topological position within Gonorynchiformes indicates a major stratigraphic gap in the record of Clupeocephala.  A similar situation occurs with the Polypteriformes, whose fossil record is rather poor and younger than that of teleosts (112–99 Ma vs. 151 Ma, respectively [7, 8]) in spite of polypteriform earlier divergence (see below).  Likewise, although the origin of the Cypriniformes total group is more ancient than that of Siluriformes, the former group has a younger fossil record (Paleocene) than the latter (Late Cretaceous). All these examples underscore important gaps in different nodes of the phylogeny, for which uniform distributions and conservatively old soft maxima are used to account for temporal uncertainty. The dagger "†" is used througout the text to denote extinct taxa.

**(1) Gnathostomata (= Root). **Node defined by the most recent common ancestor (MRCA) of *Leucoraja* and *Danio. *Hard minimum age: **†** *Elegestolepis conica*, oldest stem chondrichthyan [9]. †*Elegestolepis conica *is the oldest member of the extinct chondrichthyan family †Mongolepidae that is known from several species of the Llandovery of Central Asia [10]. Stratigraphic horizon and locality: Early Silurian; Middle-Late Llandovery, Angara-Jlim area: Niuya River outcrops and Niuya-Berresova area in the Siberian Plattform, Central Asia. Absolute age estimate: 438–426 Ma. 95% soft maximum age: 519 Ma, based on oldest deuterostome vertebrates **†** *Zhongjianichthys*, **†** *Myllokunmingia* and **†** *Haikouichthys*, Yu’anshan Member of the Heilinpu Formation (Qiongzhu Stage), Atdabanian [11]. Prior setting: 519–426 Ma, lognormal distribution, mean= 6.15, SD = 0.05 (crown calibration).

**(2) Osteichthyes. **MRCA: *Latimeria*, *Danio*. Hard minimum age: **†** *Guiyu oneiros*, oldest stem sarcopterygian [12];** **†** *Psarolepis romeri* was described by Yu [13] as a basal porolepiform, however, Zhu, Yu [14] demonstrated that it is as a basal osteichthyan.  Currently, †*Guiyi oneiros*, †*Psarolepis romeri*, and †*Achoania jarviki* are included in the *Guiyi* clade [15]. Stratigraphic horizon and locality: **†** *G. oneiros*, Kuanti Formation, Ludlow, Silurian; Eastern Yunnan, China; **†** *P. romeri*, Pridoli to Lochkovian stages, Early Devonian; Yunnan, China.Absolute age estimate: **†** *G. oneiros*, 419–418 Ma; †*P. romeri*, 417–411 Ma. 95% soft maximum age:****438–426 Ma****based on** †** *Elegestolepis conica *(see calibration 1 above). Prior setting: 438–418 Ma, exponential distribution, mean= 6.66 (crown calibration). Comments: the Devonian †*Andreolepis* has not been included in the analysis because the status of the fish is uncertain. It has been interpreted as a primitive actinopterygian, as a stem osteichthyan, and also it has been reported to show similarities to sarcopterygians and acanthodian (see [16]).

**(3) “Lobefins.” **MRCA: *Latimeria*, *Lepidosiren*. Hard minimum age: **†** *Euporosteus yunnanensis*, oldest coelacanth [14]; **†** *Uranolophus wyomingensis*, oldest stem lungfish [17]. Stratigraphic horizon and locality: **†** *E. yunnanensis*, late Pragian, Early Devonian; Yunnan, China; **†** *U. wyomingensis*, Emsian, Early Devonian; U.S.A. Absolute age estimate: **†** *E. yunnanensis*, 411–407 Ma; *P. romeri*, 407-398 Ma. 95% soft maximum age: 419–418 Ma based on** †** *G. oneiros *(see calibration 2 above). Prior setting: 419–407 Ma, exponential distribution, mean= 4.00 (crown calibration).****

**(4) Lepidosirenoidei. **MRCA: *Lepidosiren*, *Protopterus*. Hard minimum age: **†**?*Protopterus regulatus*, oldest African lungfish[18]; **†** *Lepidosiren* cf. *paradoxa*, oldest South American lungfish[19]. Stratigraphic horizon and locality: **†** *P. regulatus*, Campanian, Late Cretaceous; Egypt; **†** *L.* *paradoxa*,Maastrichtian, Late Cretaceous; Bolivia. Absolute age estimate: **†**?*P. regulatus*, 83–70 Ma; **†** *L.* *paradoxa*, 65–60 Ma. 95% soft maximum age: 416–411 Ma based on **†** *Diabolepis speratus*, Early Devonian (Lochkovian), China [20]. Prior setting: 416–70 Ma, uniform distribution (crown calibration).

**(5) Tetrapodomorpha. **MRCA: *Xenopus, Homo*. Hard minimum age: **†** *Lethiscus stocki *(lepospondyl) and **†** *Westlothiana lizziae *(aıstopod), oldest reptiliomorphs [11, 21]. Stratigraphic horizon and locality: **†** *L. stocki,* ****Wardie Shales,****near Edinburg, Scotland; **†** *W. lizziae*, East Kirkton, Scotland; both from Brigantian, Visean stage (Mississippian).  Absolute age estimate: 334–330 Ma. 95% soft maximum age: 350–346 Ma based on †*Whatcheeria *and *Pederpes*, Ivorean, North America and Europe, respectively. Prior setting: 350–330 Ma, exponential distribution, mean= 6.69 (crown calibration). This calibration follows Benton and Donoghue [11].

**(6) Metatheria. **MRCA: *Monodelphis, Homo*. Hard minimum age: **†** *Eomaia scansoria*, oldest placental with complete skeleton, hair and other soft parts [11, 22]. Stratigraphic horizon and locality: Early Cretaceous, Yixian Formation, Liaoning, China. Absolute age estimate: 124.5 Ma. 95% soft maximum age: 138–134 based on †*Vincelestes*,**Hauterivian, La Amarga Formation, Argentina. Prior setting: 139–124.5 Ma, exponential distribution, mean= 4.50 (crown calibration). This calibration follows Benton and Donoghue [11].

**(7) Human-Mouse. **MRCA: *Mus, Homo*. Hard minimum age: **†** *Heomys*, oldest putative rodent [11]. Stratigraphic horizon and locality: Danian (early Paleocene), China. Absolute age estimate: 66–62 Ma. 95% soft maximum age: 101 Ma based on oldest boreoeutherians (zalambdalestids), early Cenomanian, Khodzhakul Formation, Uzbekistan. Prior setting: 101–62 Ma, exponential distribution, mean= 12.84 (crown calibration). This calibration follows Benton and Donoghue [11].

**(8) Actinopterygii. **MRCA: *Polypterus, Danio*. Hard minimum age: **†** *Dialipina markae* and **†** *D. salgueiroensis*, oldest putative stem actinopterygians according to Schultze and Cumbaa [23], an interpretation with which we agree; **†** *Ligulalepis toombsi*[23, 24]. Stratigraphic horizon and locality: **†** *Dialipina*, Early Devonian (Lochkovian-Emsian), Siberia, Canadian Arctic; **†** *L. toombsi*, Early Devonian (Emsian-Pragian), Australia. Absolute age estimate: 415-398 Ma. 95% soft maximum age: 422–418 Ma based on **†** *Ligulalepis yunnanensis*, Late Silurian (Ludlowian), China is interpreted as an osteichthyan by Zhu and Wang [25]; 423–416 Ma,** †** *Andreolepis hedei*, Late Silurian (Ludlowian), Sweden (but see comments above). Prior setting: 423–398, exponential distribution, mean=8.35 (stem calibration). Comments: Near, Eytan [26] used a crown calibration for actinopterygians based on the stem actinopterans** **†** *Mimipiscis *and **†** *Moythomasia *(ca. 375 Ma), which may be somewhat younger than the *Polypterus-*Actinopteran split (see Xu and Gao [27]: figure 7). Instead, we use the oldest****putative total group actinopterygians as a stem calibration here and assign **†** *Mimipiscis *and **†** *Moythomasia* to the stem Actinopteri for calibration 10 (below).

**(9) Polypteriformes. **MRCA: *Erpetoichthys*, *Polypterus*. Hard minimum age: **†** *Polypterus faraou*, oldest *Polypterus *fossil [28]. Stratigraphic horizon and locality: Late Miocene, Toros-Menalla Formation; Western Djurab, Chad. Absolute age estimate: 7–5 Ma. 95% soft maximum age: 99–84Ma based on the****oldest stem polypteriform, Late Cretaceous, Sudania, Sudan [29]. Prior setting: 99–5 Ma, uniform distribution (crown calibration). Comments: Although the phylogenetic hypothesis placed the Polypteriformes as diverging at the basal actinopterygian node and predicts a divergence age of 385 My, the fossil record of the group does not even closely support such age. Nevertheless, the fossil record of Polypteriformes poses a problem not only for molecular age estimations, but it does also for morphological phylogenetic hypotheses where a major disagreement between the position of the group versus all other basal actinopterygians and their geological age is observed.  Fossil polypteriforms are only known from two Godwanan continents, South America and Africa; the oldest records are from the Albian of Brazil (112 to 99 Ma [7]) and the Cenomanian of Morocco (Kem Kem beds, Cenomanian [30, 31]). Thus, even the oldest fossil teleosts are older than fossil polypteriforms.

**(10) Actinopteri. **MRCA: *Polyodon*, *Danio*. Hard minimum age: **†** *Mimipiscis toombsi* and **†** *Moythomasia* *durgaringa*, oldest stem actinopterans[27, 32, 33]. Stratigraphic horizon and locality: Frasnian, Late Devonian; Western Australia. Absolute age estimate: 386–375 Ma. 95% soft upper bound: 415–398 Ma based on**** **†** *Dialipina markae* and **†** *D. salgueiroensis*(see calibration 8 above). Prior setting: 415–375 Ma, exponential distribution, mean= 13.7 (stem calibration). See comments under calibration 8 above.

**(11) Chondrostei. **MRCA: *Scaphirhynchus*, *Polyodon*. Hard minimum age: **†** *Protopsephurus* *liui*, oldest paddlefish [34]. Stratigraphic horizon and locality: Lower Cretaceous (Barremian-Aptian), Jianshangou beds, Lower Yixian Formation, Liaoning, China. Absolute age estimate: 125 Ma. 95% soft maximum age: 246 Ma based on **†** *Birgeria nielseni*, Sakanema Formation, Lower Triassic outcrops, northwestern Madagascar. Prior setting: 246–125 Ma, exponential distribution, mean= 40.5 (crown calibration).

**(12) Neopterygii. **MRCA: *Amia*, *Danio*. Hard minimum age: **†** *Acentrophorus varians* is interpreted here as the oldest "semionotiform", although the monophyly of the order has been questioned [35]. Stratigraphic horizon and locality: Concretionary Limestone of Late Permian, Paleozoic; Zechstein, Durham. United Kingdom (e.g., [36, 37]). Absolute age estimate: 260 Ma. 95% soft maximum age: 386–375 Ma based on** †** *Mimipiscis* *toombsi* and **†** *Moythomasia* *durgaringa*(see calibration 10 above). Prior setting: 386–260 Ma, uniform distribution.  Comments: the oldest neopterygian †*Acentrophorus* has not been included in any modern phylogenetic study or revised following modern standards. Although the situation concerning “semionotiforms” is improving due to recent research and new information (e.g., [35]), for the moment any additional comments would be premature. †*Brachydegma*, a fossil interpreted as a stem neopterygian or the oldest halecomorh by Hurley et al. [38], is excluded from consideration after examination of the material by one of us (G.A.). †*Brachydegma *lacks the synapomorphies to support such assignment (also noted by [26]), and it should be considered as a palaeosnisciform *incertae sedis, *as previously recognized [39].

**(13) Holostei. **MRCA: *Lepisosteus*, *Amia*. Hard minimum age: **†** *Watsonulus eugnathoides*, one of the oldest parasemionotid [40] and a holostean, at the base of the Halecomorpha [27, 41]. Stratigraphic horizon and locality: Induan-Olenekian boundary (Lower Triasic), Sakamena Formation, Ambilombe, Madagascar. Absolute age estimate: 246 Ma. 95% soft maximum age: 374-359 based on **†** *Kentukia hlavini*, Cleveland member, Fammenian, Late Devonian, Ohio (e.g., [42]). Prior setting: 374–246 Ma, exponential distribution, mean= 42.7 (crown calibration). Comments: the †Parasemionotidae is known by at least 10 genera from the Early Triassic of Madagascar and Greenland (e.g., [37, 43]); however, the intrarelationships of parasemionotiforms have not yet been explored. Among the pasasemionotid genera, *Watsonulus* has received more attention in recent studies [40] and has been used in several phylogenetic analyses of advanced actinopterygians (e.g., [27, 41, 44, 45]).

**(14) Elopomorpha. **MRCA: *Megalops*, *Ophichthus*. Hard minimum age: **†** *Elopsomolos frickhingeri*[8]. Stratigraphic horizon and locality: Solnhofen Limestones, Tithonian, Late Jurassic; Blumemberg, Bavaria, southern Germany [8]. Absolute age estimate: 153–149 Ma. 95% soft maximum age: 260 Ma based on** †** *Acentrophorus varians*(see calibration 12 above). Prior setting: 260–149 Ma, uniform distribution (crown calibration). †*Elopsomolos frickhingeri* is included here as the oldest member of the extant family Elopidae (Elopiformes) following the phylogenetic hypothesis of Arratia [8: fig. 22].

** (15) Albuliformes + Anguilliformes. **MRCA: *Albula*, *Ophichthus*. Hard minimum age: **†** *Baugeichthys caeruleus*, the oldest known albuliforms and the most basal one according to Filleul [46] (see also [47]). Stratigraphic horizon and locality: Hauterivian, Early Cretaceous; Massif des Bauges, France [48]. Absolute age estimate: 136 Ma. 95% soft maximum age: 216–204 Ma based on †*Pholidophorus latiusculus*, oldest known basal teleost [45]; Seefeld Formation, Norian, Late Triassic, Tirol, northern Austria [8, 49]. Prior setting: 216–136 Ma, exponential distribution, mean= 26.7 (crown calibration).

**(16) Osteoglossomorpha. **MRCA: *Hiodon*, *Pantodon*. Hard minimum age: **†** *Lycoptera davidi*, one species among many of the same age**and **†** *Yambiania wangqingica* a stem hiodontid**[45, 47, 50]. Stratigraphic horizon and locality: †*Lycoptera davidi*: Yixian Formation, Early Cretaceous; Lingyuan, Liaoning, China; †*Yambiania*: Dalazi Formation, Early Cretaceous; Wangping, Jilin,  China. Absolute age estimate: 136–130 Ma. 95% soft maximum age: 260 Ma based on** †** *Acentrophorus varians*(see calibration 12 above). Prior setting: 260–130 Ma, uniform distribution (crown calibration).

**(17) Notopteridae (total group). **MRCA: *Gymnarchus, Xenomystus*. Hard minimum age: **†** *Palaeonotopterus greenwoodi*, a stem notopterid [51]. Stratigraphic horizon and locality: Kem Kem beds,****Cenomanian, Late Cretaceous; Morocco.****Absolute age estimate: 100 Ma. 95% soft maximum age: 216–204 Ma based on **†** *Pholidophorus latiusculus *(see calibration 15 above).****Prior setting: 216–100, exponential distribution, mean= 38.7 (crown calibration).

** (18) Arapaimidae. **MRCA: *Arapaima*, *Heterotis*. Hard minimum age: **†** *Arapaima* sp., oldest Neotropical arapaimid (treated as Heterotididae indet. in Gayet and Meunier [52]). Stratigraphic horizon and locality: Maastrichtian-Danian, (Late Cretaceous),****and Santa Lucia Formation, Paleocene; Cochabamba, Bolivia [52]**.**Absolute age estimate: 73–65.5 Ma. 95% soft maximum age: 136-130 Ma based on**** **†** *Lycoptera *and **†** *Yambiania *(see calibration 16 above). Prior setting: 136–65.5 Ma, exponential distribution, mean= 23.55 (crown calibration).

**(19) Gonorynchiformes. **MRCA: *Gonorynchus*,* Chanos*. Hard minimum age: **†** *Rubiesichthys gregalis*, oldest crown gonorynchiform (family Chanidae)[3, 4]. Stratigraphic horizon and locality: Berrisian–Valangian (Early Cretaceous), Lérida, Spain. Absolute age estimate: 145.5-139 Ma. 95% soft maximum age: 216–204 Ma based on **†** *Pholidophorus latiusculus *(see calibration 17 above). Prior setting: 216–139 Ma, exponential distribution, mean= 25.7 (crown calibration).

**(20). Serrasalmidae + Hemiodontidae.** MRCA: *Pygocentrus*, *Hemiodus*. Hard minimum age: isolated Pacu teeth – oldest serrasalmid fossils [52, 53]. Stratigraphic horizon and locality: Maastrichtian-Danian (Late Cretaceous), El Molino formation, Bolivia. Absolute age estimate: 70-61 Ma. 95% soft maximum age: 97–96 Ma based on oldest characiform teeth, Cenomanian (Late Cretaceous), Sudan and Morocco [54, 55]. Prior setting: 97–61 Ma, exponential distribution, mean= 12.02 (crown calibration).

**(21) Arioidea. **MRCA: *Galeichthys*, *Gogo*. Hard minimum age: Bone and otolith fossils assignable to Ariidae sp. [56-58]. Stratigraphic horizon and locality: Late Campanian–Early Maastrichtian (Late Cretaceous), various formations in South and North America. Absolute age estimate: 73- 65.5 Ma. 95% soft maximum age: 146.5-145.5 Ma based on stem ostariophysan †*Tischlingerichthys viohli*, Late Jurassic, Germany [6]. Prior setting: 146.5–65.5 Ma, exponential distribution, mean= 27.05 (crown calibration).

**(22) Euteleostomorpha. **MRCA: *Oncorhynchus*, *Tetraodon*. Hard minimum age: †*Orthogonikleithrus hoelli*, oldest euteleost [6]. Stratigraphic horizon and locality: Solnhofen limestones, late Kimmeridgian, Late Jurassic; Ettling, Bavaria, southern Germany. Absolute age estimate: 153–149 Ma. 95% soft maximum age: 260 Ma based on** †** *Acentrophorus varians*(see calibration 12 above). Prior setting: 260–149 Ma, uniform distribution (crown calibration).

**(23) *Gasterosteus* + *Tetraodon*. **MRCA: *Gasterosteus, Tetraodon*. Hard minimum age: †*Plectocretacicus clarae*, earliest stem tetraodontiform [11, 59]. Stratigraphic horizon and locality: Cenomanian (Upper Cretaceous), Hakel, Lebanon. Absolute age estimate: 97 Ma. 95% soft maximum age: 153–149 Ma based on †*Orthogonikleithrus hoelli *(see calibration 22 above). Prior setting: 153–97 Ma, uniform distribution (crown calibration).****This calibration is modified from Benton and Donoghue [11].

**(24) Tetraodontidae. **MRCA: *Takifugu*, *Tetraodon*. Hard minimum age: †*Archaeotetraodon winterbottomi*, oldest crown tetraodontid [11, 60]. Stratigraphic horizon and locality: Lower Oligocene, Pshekhsky Horizon, Lower Maikop Formation, north Caucasus, Russia. Absolute age estimate: 32 Ma. 95% soft maximum age: 97 Ma based on †*Plectocretacicus clarae *(see calibration 23 above). Prior setting: 97–32 Ma, exponential distribution, mean= 8.01 (crown calibration).****This calibration follows Benton and Donoghue [11].

**References - Appendix 1**

1. Parham J.F., Donoghue P.C., Bell C.J., Calway T.D., Head J.J., Holroyd P.A., Inoue J.G., Irmis R.B., Joyce W.G., Ksepka D.T., et al. 2012 Best practices for justifying fossil calibrations. *Syst Biol* **61**(2), 346-359. (doi:10.1093/sysbio/syr107).

2.  Ho S.Y.W. 2009 An examination of phylogenetic models of substitution rate variation among lineages. *Biol Letters* **5**(3), 421-424. (doi:10.1098/rsbl.2008.0729).

3. Grande T., Poyato-Ariza F.J. 1999 Phylogenetic relationships of fossil and Recent gonorynchiform fishes (Teleostei: Ostariophysi). *Zool J Linn Soc* **125**(2), 197-238. (doi:10.1111/j.1096-3642.1999.tb00591.x).

4. Potayo-Ariza F.J. 1996 The phylogenetic relationships of *Rubiesichthys gregalis* and *Gordichthys conquensis* (Ostariophysi, Chanidae), from the Early Cretaceous of Spain. In *Systematics and Paleoecology* (eds. Arratia G., Viohl G.), pp. 329-348. München, Verlag Dr. Friedrich Pfeil.

5. Arratia G. 2010 The Clupeocephala re-visited: Analysis of characters and homologies. *Revista de Biologia Marina & Oceanografia* **45**(Special issue), 635-657.

6. Arratia G. 1997 Basal teleosts and teleostean phylogeny. *Palaeo Ichthyologica* **7**, 1-168.

7. Dutra M.F.A., Malabarba M.C. 2001 Peixes do Albiano-Cenomaniano do Grupo Itapecuru no estado do Maranhão, Brasil. In *O Cretáceo da Bacia de São Luis-Grajaú* (eds. Rossett D.F., Góes A.M., Truckenbrodt T.), pp. 191-208, MPEQ.

8. Arratia G. 2000 Remarkable teleostean fishes from the Late Jurassic of southern Germany and their phylogenetic relationships. *Mitteilungen aus dem Museum für Naturkunde, Berlin, Geowissenschaftliche Reihe* **3**, 137-179.

9. Karatajüte-Talimaa V., Predtechenskyi N. 1995 The distribution of the vertebrates in the Late Ordovician and early Silurian palaeobasins of the Siberian Plattform. In *Etudes surles Vertébrés inférieurs (VIIe Symposium International, Miguasha, Québec,1991)* (eds. Arsenault M., Lelièvre H., Janvier P.), pp. 39–55. Paris, Bulletin Muséum National d’Histoire naturelle.

10. Karatajüte-Talimaa V. 1995 The Mongolepidae: Scale structure and systematics position. *Geobios* **19**, 35-37.

11. Benton M.J., Donoghue P.C.J. 2007 Paleontological evidence to date the tree of life. *Mol Biol Evol* **24**(1), 26-53. (doi:Doi 10.1093/Molbev/Msl150).

12. Zhu M., Zhao W., Jia L., Lu J., Qiao T., Qu Q. 2009 The oldest articulated osteichthyan reveals mosaic gnathostome characters. *Nature* **458**, 469–474. (doi: doi:10.1038/nature07855).

13. Yu X. 1998 A new porolepiform-like fish, *Psarolepis romeri*, gen. et sp. nov. (Sarcopterygii, Osteichthyes) from the Lower Devonian of Yunnan, China. *J Vertebr Paleontol* **18 **(2), 261–274.

14. Zhu M., Yu X., Lu J., Qiao T., Zhao W., Jia L. 2012 Earliest known coelacanth skull extends the range of anatomically modern coelacanths to the Early Devonian. *Nature* **3**, 772. (doi:10.1038/ncomms1764).

15. Qiao T., Zhu M. 2010 Cranial morphology of the Silurian sarcopterygian *Guiyu oneiros* (Gnathostomata: Osteichthyes). *Science China Earth Sciences* **53**(12), 1836-1848.

16. Cunningham J.A., Rucklin M., Blom H., Botella H., Donoghue P.C. 2012 Testing models of dental development in the earliest bony vertebrates, *Andreolepis* and *Lophosteus*. *Biol Letters* **8**(5), 833-837. (doi:doi: 10.1098/rsbl.2012.0357).

17. Denison R.H. 1968 Early Devonian lungfishes from Wyoming, Utah, and Idaho. *Fieldiana, Geology* **17**(4), 410-413.

18. Schall S. 1984 Oberkretazische Osteichthyes (Knochen-fische) aus dem Bereich von Bahariya und Kharga, Aegypten, und ihre Aussagen zur Paläkologie und Stratigraphie. . *Berliner Geowissenschaftliche Abhandlungen* **A 53**, 1-79.

19. Schultze H.-P. 1991 Lungfish from El Molino (late Cretaceous) and Santa Lucía (Early Paleocene) formations in south central Bolivia. In *Fósiles y Facies de Bolivia* (ed. Suárez-Soruco R.), pp. 441-448, Revista Técnica de Yacimientos Petrolíferos Fiscales Bolivianos.

20. Chang M.M., Yu X. 1984 Structure and phylogenetic significance of *Diabolichthys speratus* gen. et sp. nov., a new dipnoan-like form from the Lower devonian of eastern Yunan. *Proc Linn Soc N S W* **107**, 171-184.

21. Ruta M., Coates M., Quicke D. 2003 Early tetrapod relationships revisited. *Biol Rev (Camb)* **78**(2), 251-345.

22. Ji Q., Luo Z.-X., Yuan C.-X., Wible J.R., Zhang J.-P., Georgi J.A. 2002 The earliest known eutherian mammal. *Nature* **416**(6883), 816-822.

23. Schultze H.-P., Cumbaa S.L. 2001 *Dialipina* and the characters of basal actinopterygians. In *Major events in Early Vertebrate Evolution, Paleontology, Phylogeny, Genetics and Development* (ed. Ahlberg P.E.), pp. 315-332. London and New York., Systematic Association Special Volume - Taylor & Francis.

24. Schultze H.-P. 1968 Palaeoniscoidea-Schuppen aus dem Unterdevon Australiens und Kanadas dem Mitteldevon Spitzbergens *Bulletin of the British Museum of Natural History* **16**, 343-368.

25. Zhu M., Wang J.-Q. 2000 Silurian vertebrate assemblages of China. *Courier Forsch-Institut Senckenberg* **223**(161-168).

26. Near T.J., Eytan R.I., Dornburg A., Kuhn K.L., Moor J.A., Davis M.P., Wainwright P.C., Friedman M., Smith W.L. 2012 Resolution of ray-finned fish phylogeny and timing of diversification. *PNAS*. (doi:10.1073/pnas.1206625109).

27. Xu G.-H., Gao K.-Q. 2011 A new scanilepiform from the Lower Triassic of northern Gansu Province, China, and phylogenetic relationships of non-teleostean Actinopterygii. *Zool J Linn Soc* **161**(3), 595-612. (doi:10.1111/j.1096-3642.2010.00645.x).

28. Otero O., Andossa L., Vignaud P., Brunet M. 2006 A new polypterid fish: *Polypterus faraou* sp. nov. (Cladistia, Polypteridae) from the Late Miocene, Toros-Menalla, Chad. *Zool J Linn Soc* **146**(2), 229-237.

29. Werner C., Gayet M. 1997 New fossil polypterids from the Cenomanian of Sudan. An evidence of their high diversity in the early Late Cretaceous. *Cybium* **6**, 67-81.

30. Dutheil D.B. 1999 An overview of the freshwater fish fauna from the Kem Kem beds (Late Cretaceous: Cenomanian) of southeast Morocco. In *In: Mesozoic Fishes 2 – Systematics and Fossil Record; *(eds. Arratia G., Schultze H.-P.), pp. 553-563. München, Verlag Dr. F. Pfeil.

31. Gayet M., Meunier F.J. 2002 Diversification of Polypteriformes and special comparisosn with Lepisosteiformes. *Palaeontology (Oxford)* **45**(2), 361-376.

32. Gardiner B.G. 1993 Osteichthyes: Basal Actinopterygians. In *Fossil Record II* (ed. Benton M.J.), pp. 611-619. London, Chapman and Hall.

33. Gardiner B.G., Schaeffer B. 1989 Interrelationships of lower actinopterygian fishes. *Zool J Linn Soc* **97**(2), 135-187. (doi:10.1111/j.1096-3642.1989.tb00550.x).

34. Grande L., Jin F., Yabumoto Y., Bemis W.E. 2002 †*Protopsephurus liui*, a well-preserved primitive paddlefish (Acipenseriformes: polyodontidae) from the lower cretaceous of China. *J Vertebr Paleontol* **22**, 209–237.

35. López-Arbarello A. 2012 Phylogenetic Interrelationships of Ginglymodian Fishes (Actinopterygii: Neopterygii). *PLoS ONE* **7**(7), e39370. (doi:10.1371/journal.pone.0039370).

36. Kirkby J.W. 1862 On the remains of fish and plants from the upper limestone of the Permian series of Durham. *The Annals and Magazine of Natural History* **9**, 267-269.

37. Lehman P. 1966 Actinopterygii. In *Traité de Paléontologie Tomo IV, L' origine des Vertébrés Leur expansion dans les eaux douces et le milieu marin, - Agnathes - Placodermes - Elasmobranches - Actinoptérygiens -Crossoptérygiens - Dipneustes* (ed. Piveteau J.), pp. 1-242. Paris, Masson et Cie.

38. Hurley I.A., Mueller R.L., Dunn K.A., Schmidt E.J., Friedman M., Ho R.K., Prince V.E., Yang Z.H., Thomas M.G., Coates M.I. 2007 A new time-scale for ray-finned fish evolution. *Proc R Soc B-Biol Sci* **274**(1609), 489-498.

39. Dunkle D.H. 1939 A new Palaeoniscid fish from the Texas Permian. *American Journal of Sciences* **237**, 262-274.

40. Olsen P.E. 1984 The skull and pectoral girdle of the parasemionotid fish *Watsonulus eugnathoides* from the Early Triassic Sakamena Group of Madagascar, with comments on the relationships of the holostean fishes. *J Vertebr Paleontol* **4**, 481–499.

41. Grande L., Bemis W.E. 1998 A comprehensive phylogenetic study of amiid fishes (Amiidae) based on comparative skeletal anatomy. An empirical search for interconnected patterns of natural history. *Journal of Vertebrate Paleontology (Memoir 4, supplement)* **18**(1), 690.

42. Carr R.K., Jackson G.L. 2008 The vertebrate fauna of the Cleveland Member (Famennian) of the Ohio Shale. *Ohio Geological Survey, Guidebook* **22 **1-17.

43. Beltan L. 1996 Overview of systematics, paleobiology, and paleoecology of Triassic fihes of northwest Madagascar. In *Mesozoic Fishes – Systematics and Paleoecology* (eds. Arratia G., Viohl G.), pp. 479-500. München, Verlag Dr. F. Pfeil.

44. Arratia G. 1999 The monophyly of Teleostei and stem-group teleosts. Consensus and disagreements. In *Mesozoic Fishes 2 – Systematics and Fossil Record* (eds. Arratia G., Schultze H.P.), pp. 265-334. München, Verlag Dr. F. Pfeil.

45. Arratia G. 2000 New teleostean fishes from southern Germany and the systematic problems concerning the 'pholidophoriforms'. *Paläontologische Zeitschrift* **74**, 113-143.

46. Filleul A. 2000 Analyse critique des synapomorphies des Elopomorphes et analyse phylogénétique du groupe. *Cybium* **4**(3), 75-83.

47. Arratia G. 2010 Critical analysis of the impact of fossils on teleostean phylogenies, especially that of basal teleosts. In *Morphology, Phylogeny and Paleobiogeography of Fossil Fishes* (eds. Elliott D.K., Maisey J.G., Yu X., Miao D.S.), pp. 247-274. München, Germany, Verlag Dr. Friedrich Pfeil.

48. Filleul A. 2000 *Baugeichthys caeruleus*, gen. et sp. nov., a new albuliform fish from the Hauterivian of the Massif des Bauges (France). *Journal of Vertebrate Paleontolology* **20**, 637-644.

49. Arratia G. 2004 Mesozoic halecostomes and the early radiation of teleosts. In *Mesozoic Fishes 3 – Systematics, Paleoenvironments and Biodiversity* (eds. Arratia G., Tintori A.), pp. 279-315. München, Verlag Dr. F. Pfeil.

50. Li G.-Q., Wilson M.V.H. 1999 Early divergence of Hiodontiformes sensu stricto in East Asia and phylogeny of some Late Mesozoic teleosts from China. In *Mesozoic Fishes 2 – Systematics and Fossil Record* (eds. Arratia G., Schultze H.-P.), pp. 369-384. München Verlag Dr. Friedrich Pfeil.

51. Cavin L., Forey P. 2001 Osteology and systematic affinities of *Palaeonotopterus greenwoodi* Forey 1997 (Telesotei: Osteoglossomorpha). *Zool J Linn Soc* **133**, 25-52.

52. Gayet M., Meunier F.J. 1998 Maastrichtian to early Late Paleocene Freshwater osteichthyes of Bolivia: Additions and comments. In *Phylogeny and Classification of Neotropical Fishes* (eds. Malabarba L.R., Reis R.E., Vari R.P., Lucena Z.M.S., Lucena C.A.S.), pp. 85-110. Porto Alegre, EDIPUCRS.

53. Dahdul W.M. 2007 Phylogenetics and diversification of the Neotropical Serrasalminae (Ostariophysi: Characiformes). Pennsylvania, University of Pennsylvania.

54. Malabarba L.R., Malabarba M.C. 2010 Biogeography of Characiformes: an evaluation of the available information of fossil and extant taxa. In *Origin and Phylogenetic Interrelationships of Teleosts* (eds. Nelson J.S., Schultze H.P., Wilson M.V.H.), pp. 317-336. München, Germany, Verlag Dr. Friedrich Pfeil.

55. Werner C. 1994 Die kontinentale Wirbeltierfauna aus der unteren Oberkreide des Sudan (Wadi Milk Formation). *Geowiss* **13**, 221-249.

56. Gayet M., Meunier F.J. 2003 Paleontology and palaeobiogeography of catfishes. In *Catfishes* (eds. Arratia G., Kapoor B.G., Chardon M., Diogo R.), pp. 491–522. Enfield, NH, Science Publishers.

57. Lundberg J.G., Sullivan J.P., Rodiles-Hernández R., Hendrickson D.A. 2007 Discovery of African roots for the Mesoamerican Chiapas catfish, *Lacantunia enigmatica*, requires an ancient intercontinental passage. *Proc Acad Nat Sci Phila* **156**, 39-53.

58. Betancur-R R., Ortí G., Stein A.M., Marceniuk A.P., Alexander Pyron R. 2012 Apparent signal of competition limiting diversification after ecological transitions from marine to freshwater habitats. *Ecol Lett* **15**(8), 822-830. (doi:10.1111/j.1461-0248.2012.01802.x).

59. Tyler J.C., Sorbini L. 1996 New superfamily and three new families of tetraodontiform fishes from the Upper Cretaceous: the earliest and most morphologically primitive plectognaths. *Smithson Contrib Paleobiol* **82**, 1–59.

60. Santini F., Tyler J.C. 2003 A phylogeny of the families of fossil and extant tetraodontiform fishes (Acanthomorpha, Tetraodontiformes), Upper Cretaceous to Recent. *Zool J Linn Soc* **139**, 565–617.

## Appendix 2

### Sequence data present in this study

**Table 1:** Sequences included in this study.  1=sequenced for this study, x=obtained from GenBank or Ensemble database, 0=absent.  / indicates sequences obtained from two congernic species.

* *	*apc2*	*btbd7*	*enc1*	*ficd*	*gtdc2*	*gpr85*	*kbtbd4*	*kiaa-l*	*myh6*	*panx2*	*plagl2*	*ptchd4*	*rag1*	*rag2*	*ryr3*	*sacs-l*	*snx33*	*tbcc-l*	*tbr1b*	*zic1*	16S	TOTAL
*Callorhinchus milii*	x	x	x	x	x	x	x	x	0	x	x	x	x	x	x	x	x	x	0	0	x	18
*Leucoraja erinacea*	x	x	x	x	x	x	x	x	0	x	x	x	x	x	x	x	x	x	0	x	x	19
*Xenopus tropicalis*	0	x	x	x	x	x	x	x	x	x	x	x	x	x	x	x	x	x	x	x	x	20
*Monodelphis domestica*	x	x	x	x	x	x	x	0	x	x	x	x	x	x	x	x	x	x	x	x	x	20
*Mus musculus*	x	x	x	x	x	x	x	x	x	x	x	x	x	x	x	x	x	x	x	x	x	21
*Homo sapiens*	x	x	x	x	x	x	x	x	x	x	x	x	x	x	x	x	x	x	x	x	x	21
*Protopterus_aethiopicus/annectens*	0	0	0	1	0	1	0	0	0	0	0	0	x	x	0	1	0	0	0	0	x	6
*Lepidosiren paradoxa*	0	0	0	0	0	1	0	0	0	0	0	0	x	x	0	0	0	0	0	0	x	4
*Latimeria chalumnae*	x	x	0	x	0	x	x	x	x	x	0	0	x	x	0	x	0	x	0	0	x	13
*Polypterus senegalus*	0	0	0	0	1	x	1	1	0	1	1	1	1	x	1	1	0	0	1	x	x	14
*Erpetoichthys calabaricus*	1	0	0	0	0	1	1	0	0	1	0	0	1	0	0	1	0	0	0	1	x	8
*Polyodon spathula*	0	0	1	1	0	x	1	1	1	0	1	1	1	x	1	1	0	1	0	x	x	15
*Acipenser fulvescens*	0	1	0	1	0	1	1	1	0	0	0	0	1	0	0	0	0	1	0	1	1	9
*Scaphirhynchus albus*	0	1	0	0	0	1	1	1	0	1	0	0	x	1	0	0	0	1	0	1	1	10
*Lepisosteus osseus*	1	1	0	1	0	x	1	1	x	1	1	0	1	x	1	1	0	1	0	x	x	16
*Lepisosteus platostomus*	0	1	0	1	0	1	1	1	1	1	0	0	1	0	0	1	0	1	0	1	1	12
*Amia calva*	0	1	0	1	1	x	1	1	x	0	1	1	1	x	1	1	1	0	1	x	x	17
*Hiodon alosoides*	0	0	1	1	0	x	1	1	x	1	1	1	1	x	1	1	1	1	1	x	x	18
*Xenomystus nigri*	0	1	0	1	0	1	1	1	0	0	0	0	1	x	0	1	0	1	0	1	x	11
*Gnathonemus petersii*	0	0	0	1	0	1	1	1	1	0	0	0	1	x	0	0	0	1	0	1	x	10
*Gymnarchus niloticus*	0	1	0	0	0	1	1	1	1	1	0	0	1	x	0	1	0	0	0	1	x	11
*Osteoglossum bicirrhosum*	0	0	1	0	0	x	1	1	0	0	1	0	x	x	0	0	0	1	1	x	x	11
*Pantodon buchholzi*	0	1	0	0	0	1	1	1	0	1	0	0	1	x	0	0	0	0	0	1	x	9
*Arapaima gigas*	0	1	0	1	0	1	1	1	0	1	0	0	1	x	0	1	0	1	0	1	x	12
*Heterotis niloticus*	0	1	0	0	0	1	0	1	0	1	0	0	1	x	0	1	0	1	0	1	1	10
*Elops saurus*	0	0	1	0	0	x	1	1	x	1	1	1	1	0	1	0	1	1	1	x	x	16
*Megalops atlanticus*	0	0	0	1	0	1	0	1	1	1	0	0	x	0	0	0	0	0	0	0	x	7
*Albula vulpes*	0	0	1	1	0	0	1	1	x	1	0	0	x	1	0	1	0	1	0	x	1	12
*Halosauropsis macrochir*	1	0	0	1	0	1	1	1	1	1	0	0	1	0	0	1	0	1	0	1	1	12
*Notacanthus chemnitzii*	1	0	0	1	0	0	1	1	1	1	0	0	1	0	0	1	0	1	0	1	x	11
*Echidna nebulosa*	0	0	0	0	0	0	1	1	1	1	0	0	1	1	0	0	0	0	0	0	1	7
*Anguilla rostrata*	1	0	1	1	0	0	1	1	x	1	0	1	1	0	0	1	1	1	0	x	x	14
*Muraenesox cinereus*	1	0	0	1	0	1	1	1	0	1	0	0	1	0	0	1	0	0	0	1	1	10
*Nemichthys scolopaceus*	1	0	0	1	0	1	1	1	1	1	0	0	1	0	0	1	0	1	0	0	1	11
*Ophichthus cephalozona*	0	0	0	1	0	1	1	1	1	1	0	0	1	0	0	1	0	0	0	1	1	10
*Dorosoma cepedianum*	1	0	0	0	1	x	1	1	x	1	0	0	x	x	1	0	0	1	0	x	x	13
*Engraulis eurystole*	1	0	0	0	0	1	1	1	0	1	0	0	x	x	0	1	0	0	0	1	x	10
*Chriocentrus dorab*	0	0	1	0	1	1	0	0	1	0	1	0	x	1	1	0	0	0	0	1	0	9
*Pellona flavipinnis*	0	0	1	0	1	x	1	1	x	1	1	1	x	x	1	1	0	1	0	x	x	16
*Chanos chanos*	0	1	1	0	0	x	1	1	x	1	1	1	x	1	0	1	0	1	1	x	x	16
*Gonorynchus forsteri*	0	0	0	0	0	0	0	0	1	0	0	0	1	0	0	0	0	0	0	1	0	3
*Danio rerio*	x	x	x	x	x	x	x	x	x	x	x	x	x	x	x	x	x	x	x	x	x	21
*Notemigonus crysoleucas*	0	0	1	0	1	1	0	0	1	0	1	1	1	0	1	0	0	0	1	1	0	10
*Semotilus atromaculatus*	0	0	1	0	1	1	0	0	1	0	1	1	0	0	1	0	1	0	1	1	0	10
*Apteronotus albifrons*	0	0	1	0	0	1	0	0	0	0	1	1	1	0	0	0	1	0	1	1	0	8
*Hemiodus immaculatus/gracilis*	0	0	0	0	0	0	0	0	1	0	0	0	1	1	0	0	0	0	0	0	0	3
*Pygocentrus nattereri*	1	0	1	0	0	x	1	1	x	1	1	1	1	1	1	1	1	1	0	0	1	16
*Galeichthys peruvianus*	0	0	0	0	0	0	0	0	1	0	0	0	1	1	0	0	0	0	0	0	0	3
*Gogo arcuatus*	0	0	0	0	0	0	0	0	1	0	0	0	1	1	0	0	0	0	0	0	0	3
*Ictalurus punctatus*	0	1	1	0	1	x	1	0	x	1	1	1	1	1	1	1	1	0	1	x	x	17
*Argentina sialis*	0	0	1	1	1	1	0	1	1	1	0	1	1	0	1	0	1	0	1	1	0	13
*Esox lucius*	0	0	1	0	1	1	0	1	1	1	1	1	1	0	1	0	1	0	0	1	1	13
*Oncorhynchus mykiss/nerka*	0	0	1	0	1	1	0	1	1	1	1	1	1	1	1	0	1	0	1	1	x	15
*Stomias boa*	0	0	1	0	1	1	0	1	1	1	0	1	1	0	0	0	1	0	1	1	0	11
*Thaleichthys pacificus*	0	0	1	1	1	1	0	0	1	1	0	1	1	0	0	0	1	0	1	1	0	11
*Gadus morhua*	x	x	x	x	x	x	x	x	x	x	x	x	x	0	0	x	x	x	0	x	x	18
*Oreochromis niloticus*	x	x	x	x	x	x	x	x	x	x	x	x	x	x	x	x	x	x	x	x	x	21
*Oryzias latipes*	x	x	x	x	x	x	x	x	x	x	x	x	x	x	x	x	x	x	x	x	x	21
*Gasterosteus aculeatus*	x	x	x	x	x	x	x	x	x	x	x	x	x	x	x	x	x	x	x	x	x	21
*Takifugu rubripes*	x	x	x	x	x	x	x	x	x	x	x	x	x	x	x	x	x	x	x	x	x	21
*Tetraodon nigroviridis*	x	x	x	x	x	x	x	x	x	x	x	x	x	x	x	x	x	x	x	x	x	21
TOTAL	23	26	32	35	26	53	44	47	45	45	30	31	60	41	28	38	26	35	24	50	50	789
